# Oxygen Uptake in Maximal Effort Constant Rate and Interval Running

**DOI:** 10.1155/2013/680326

**Published:** 2013-09-01

**Authors:** Daniel Pratt, Brendan J. O'Brien, Bradley Clark

**Affiliations:** School of Health Sciences, University of Ballarat, Mt Helen, Ballarat, VIC 3353, Australia

## Abstract

This study investigated differences in average $\dot{\text{V}}{\text{O}}_{2}$ of maximal effort interval running to maximal effort constant rate running at lactate threshold matched for time. The average $\dot{\text{V}}{\text{O}}_{2}$ and distance covered of 10 recreational male runners (${\dot{\text{V}}{\text{O}}_{2}}_{\text{ max}}$: 4158 ± 390 mL*·*min^−1^) were compared between a maximal effort constant-rate run at lactate threshold (CRLT), a maximal effort interval run (INT) consisting of 2 min at ${\dot{\text{V}}{\text{O}}_{2}}_{\text{ max}}$ speed with 2 minutes at 50% of $\dot{\text{V}}{\text{O}}_{2}$ repeated 5 times, and a run at the average speed sustained during the interval run (CR submax). Data are presented as mean and 95% confidence intervals. The average $\dot{\text{V}}{\text{O}}_{2}$ for INT, 3451 (3269–3633) mL*·*min^−1^, 83% ${\dot{\text{V}}{\text{O}}_{2}}_{\text{ max}}$, was not significantly different to CRLT, 3464 (3285–3643) mL*·*min^−1^, 84% $\dot{\text{V}}{\text{O}}_{\text{2 max}}$, but both were significantly higher than CR sub-max, 3464 (3285–3643) mL*·*min^−1^, 76% ${\dot{\text{V}}{\text{O}}_{2}}_{\text{ max}}$. The distance covered was significantly greater in CLRT, 4431 (4202–3731) metres, compared to INT and CR sub-max, 4070 (3831–4309) metres. The novel finding was that a 20-minute maximal effort constant rate run uses similar amounts of oxygen as a 20-minute maximal effort interval run despite the greater distance covered in the maximal effort constant-rate run.

## 1. Introduction

The principal objective of endurance training is to evoke supracompensation in the physiological systems restraining the maximal sustainable competition speed. The physiological systems most noted for regulating the speed of an endurance runner are the convective supply of oxygen to the muscles and the rate at which oxygen can be metabolized in the muscles to resynthesize adenosine triphosphate (ATP) [1]. It is proposed that the training strategy that sustains the highest oxygen use ($\dot{\text{V}}{\text{O}}_{2}$) for the longest is the most effective strategy to improve running performance [2]. $\dot{\text{V}}{\text{O}}_{2}$ is typically assessed by the minute rate of pulmonary oxygen uptake during running [3]. The training strategies used by athletes can be broadly classed into constant rate running or interval running, where interval involves higher speeds of running interspersed with slower “recovery” speeds. 

Interval running evokes a greater total $\dot{\text{V}}{\text{O}}_{2}$ than constant rate running [4] when the average speed of the treatments is controlled. Additionally, Daussin et al. [5] revealed that interval training over several weeks' results in greater gains in cycling performance, metabolic, and cardiorespiratory adaptation. However, any assumption interval training is superior to constant rate training may be erroneous and an artefact of the research design. Generally, the work (run speed or cycle wattage) completed in a specific time frame has been controlled in the experimental treatments to ensure that comparisons in training adaptation are not biased by differences in work of the training strategies. However, by controlling work in the constant rate to interval training, the sustainable constant rate speed/wattage is less than the maximal sustainable speed (i.e., the constant rate training is still “submaximal”). For example, in O'Brien et al.'s [4] investigation, it was reported that interval running used more oxygen than constant rate running; however, the participants performed the constant rate run at a speed equivalent to the interval running mean speed, estimated to be only 75%  ${\dot{\text{V}}\text{O}}_{2\max}$, which was most likely below the lactate threshold or the fastest speed able to be sustained continuously by the runner. Consequently, equalising speed or work of the interval or constant rate runs may mask the optimal training strategy for athletes, and for all practical purposes, matching maximal effort over a duration recommended to improve cardiorespiratory fitness is more appropriate. The ACSM currently recommends that 20 minutes of exercise is required to improve cardiorespiratory fitness [6]. Therefore, we aim to compare the total $\dot{\text{V}}{\text{O}}_{2}$ of a maximal effort interval run to a maximal effort constant rate run, matched for time, 20 minutes.

## 2. Methods

### 2.1. General Design of Study

This study is a quantitative study with a crossover design where participants in random sequence completed constant rate and interval training treadmill running at their individual-perceived maximal effort speed to investigate which strategy results in greater pulmonary oxygen uptake per minute ($\dot{\text{V}}{\text{O}}_{2}$).

### 2.2. Participants

Ten “fit” males (${\dot{\text{V}}\text{O}}_{2\max}\;\;4158\pm 390$ mL·min^−1^) were tested through recruitment via the university and personal contacts. The participants were aged from 18 to 40 years old.

### 2.3. Experimental Protocol

Each participant included competed two preliminary running tests and three experimental runs which were compared.

 Preliminary test 1: an initial maximal treadmill test to establish ${\dot{\text{V}}\text{O}}_{2\max}$ and the speed at which it is achieved.
 Preliminary test 2: a 5 km run time trial to estimate the maximal constant-rate speed approximating lactate threshold.
 Experimental test 1: a maximal effort interval treadmill run consisting of 5 × 2 minute intervals at the speed corresponding to ${\dot{\text{V}}\text{O}}_{2\max}$  ($\text{s}{\dot{\text{V}}\text{O}}_{2\max}$) during the high periods and 5 × 2 minute intervals at 0.5 $\text{s}{\dot{\text{V}}\text{O}}_{2\max}$.
 Experimental test 2: a maximal effort constant rate treadmill run at the highest velocity that could be sustainable speed over 20 minutes (constant rate approximating lactate threshold run). This was determined from the speed calculated from a 5 km time trial performed on a public park.
 Experimental test 3: a constant rate treadmill run at a speed determined from the average speed of the interval protocol used in Experimental test 1.

### 2.4. Experimental Procedure

The initial preliminary test of ${\dot{\text{V}}\text{O}}_{2\max}$  and its corresponding speed was conducted in an exercise physiology laboratory. Prior to the ${\dot{\text{V}}\text{O}}_{2\max}$ test, participants were fitted with a two-way breathing valve (Hans Rudolph, USA), and expired air was collected into an online metabolic system (Moxus, USA) to analyse $\dot{\text{V}}{\text{O}}_{2}$. The metabolic system was calibrated before each test using ambient air and gas of known composition. The ${\dot{\text{V}}\text{O}}_{2\max}$ test commenced at 9 km·h^−1^at a gradient of 1%, and treadmill speed was increased by 1 km·h^−1^ every 2 minutes until volitional exhaustion. ${\dot{\text{V}}\text{O}}_{2\max}$ was determined as the highest 60-second $\dot{\text{V}}{\text{O}}_{2}$ value recorded during the test. Within a week of ${\dot{\text{V}}\text{O}}_{2\max}$ determination, the 5 km time trial test was performed on flat terrain at a public park.

After the two preliminary tests, the participants completed the interval and the constant rate runs on the exercise physiology laboratory treadmill on separate days in random sequence. The experimental runs were preceded by a standardized 5-minute warm-up run on the treadmill at 60% of ${\dot{\text{V}}\text{O}}_{2\max}$ followed by 2-minute rest. To control the confounding variables of diet, hydration, and fatigue, the participants were asked to consume 8–10 g of carbohydrate per kg of body weight, drink adequate fluid to maintain hydration, and sleep a minimum of 7 hours the night prior to testing. 

During all experimental treadmill runs, expired air was collected for metabolic analysis as per the initial maximal test. The $\dot{\text{V}}{\text{O}}_{2}$ was recorded continuously in 30-second segments during each 20-minute run to determine the average $\dot{\text{V}}{\text{O}}_{2}$. To confirm if the runs were the highest sustainable perceived effort for 20 minutes, each participant initially ran at the speed determined from the preliminary tests. The constant-rate run at lactate threshold was initially attempted by all participants at the speed determined from the 5 km time trial performed at the public park. The interval run on the treadmill was initially attempted at the final treadmill speed from the ${\dot{\text{V}}\text{O}}_{2\max}$ test, with the recovery periods set at 50% of the final treadmill speed. If the participant completed the 20 minutes in either the interval or constant rate run at lactate threshold, they undertook the run on another day at a higher speed. If the participant could not complete the 20-minute run, they ran on another day at a lower speed. The increase or decrease in speed was subjectively determined by the participant to their projected perception of what they felt could be a maximal effort. Originally, we planned to alter increments or decrements in speed by 0.2 km·h^−1^, although it quickly became apparent that some individuals felt 0.2 km·h^−1^ changes would be too “easy” or “not enough,” so we decided it was more appropriate for the individual to determine their own speed adjustments to establish a maximal perceived effort. The number of runs to determine a maximal effort was capped at three attempts for ethical and time constraints. The fastest speed able to be sustained for 20 minutes by the participant was used in the statistical analysis. The mean final treadmill speed from the initial $\dot{\text{V}}{\text{O}}_{2}$ max test was 16.1 km·h^−1^, and the mean final effort sustainable interval speed was 16.3/8.15 km·h^−1^. The mean time of the 5 km time was 14 km·h^−1^ although this was not tolerated well on the laboratory treadmill by the majority of participants, with the mean maximal effort speed being 13.4 km·h^−1^. 

### 2.5. Statistical Analyses

Differences in average $\dot{\text{V}}{\text{O}}_{2}$ and mean distance covered between the three run protocols were analysed using linear mixed models (LMMs), with “type” as a fixed effect. Two error covariance structures were tested—independence (zero covariance) and repeated measures structures (compound symmetry—constant covariance between each pair of types). Models were compared using likelihood ratio tests, which confirmed the compound symmetry structure. Paired *t*-tests with Bonferroni correction were conducted to determine the significance of pairwise differences. Assumptions of normality and homogeneous variance of errors were tested by graphical display and analysis of residuals and found to be normally distributed. Significance was assumed at the 5% level. All statistical analyses were carried out using SPSS Version 19.

## 3. Results

The mean$\;\dot{\text{V}}{\text{O}}_{2}$ of the three running protocols is presented in Table 1.

The mean $\dot{\text{V}}{\text{O}}_{2}$ and $\dot{\text{V}}{\text{O}}_{2}/{\dot{\text{V}}\text{O}}_{2\max}$ (%) were similar between the interval and constant rate at lactate threshold runs but were significantly greater in both maximal effort runs compared to submaximal constant rate run. The distance covered during the constant rate at lactate threshold run was significantly greater (*P* < 0.05) than the distance covered during the maximal Interval and submaximal constant rate runs. 

## 4. Discussion

The purpose of this study is to elucidate whether constant-rate running has the potential to equal or exceed the oxygen uptake of maximal effort interval training by comparing the $\dot{\text{V}}{\text{O}}_{2}$ between maximal interval and constant rate run efforts, matched for duration of running, 20 minutes. The major finding of this study is that interval running and constant-rate running use similar amounts of oxygen when performed at the maximal sustainable speed for an individual.

Both maximal interval and the constant rate at lactate threshold run resulted in a significantly greater (*P* < 0.05) mean $\dot{\text{V}}{\text{O}}_{2}$ consumption compared to the submaximal constant rate run (3451 and 3434 versus 3141 mL·min^−1^). This difference can be explained by the higher average relative intensity of the exercise of the maximal interval and the constant rate at lactate threshold runs compared to the submaximal constant rate run (83% and 84% versus 76% $\dot{\text{V}}{\text{O}}_{2}/{\dot{\text{V}}\text{O}}_{2\max}$ (%)). The similar oxygen requirement of both maximal running strategies challenges the assumption that interval training is a superior form of training to maximal effort constant rate training. Previous studies report interval training results in greater total $\dot{\text{V}}{\text{O}}_{2}$ of a workout compared to constant-rate training [2, 4, 7, 8] and Daussin et al. [5] clearly showed physiological adaptations were superior after interval training. However, Billat et al. [2] and Demarie et al. [7] used a very high intensity for the constant rate run (approximately 92% of $\text{v}{\dot{\text{V}}\text{O}}_{2\max}$) that did not allow exercise to be sustained for a duration from (eight to ten minutes) normally sustained in typical endurance athlete training (at least 20 minutes). On the other hand, the studies by O'Brien et al. [4] and Daussin et al. [5] performed the constant rate run at a submaximal intensity (72% ${\dot{\text{V}}\text{O}}_{2\max}$ and approximately at 60% ${\dot{\text{V}}\text{O}}_{2\max}$,  resp.) that does not drive $\dot{\text{V}}{\text{O}}_{2}$ near ${\dot{\text{V}}\text{O}}_{2\max}$. The significance of our finding is that when matched for duration, constant rate approximating lactate threshold training places similar aerobic “load” as maximal interval training and therefore may be equally effective in enhancing running performance. Future research is required to compare a constant rate at lactate threshold training versus maximal effort interval training performed over several weeks to determine if any has a superior outcome on time trial performance. Interestingly, the constant rate at lactate threshold running resulted in a significantly greater distance being covered than interval running (4470 versus 4070 m), despite using similar amounts of oxygen. Consequently, maximal effort constant-rate running is a more effective and more economic strategy to cover a set distance in 20 minutes. The most likely explanation of the greater oxygen use in interval running is the excess postoxygen consumption that accumulates after each of the 2 min high intensity efforts. The excess post oxygen consumption is attributable to a number of factors but most likely is consequential to greater need for phosphate creatine restoration [9] and sodium/potassium regulation associated with repeated high intensity efforts that have a high anaerobic reliance [10].

### 4.1. Limitations

A limitation of this study was the determination of maximal effort that was capped at three attempts for each of the interval and constant-rate at lactate threshold runs. In the ideal experimental model, we would have requested participants to report more frequently to the laboratory to pinpoint maximal effort more precisely (i.e., any further increase in treadmill speed would lead to failure to complete the 20-minute run). Our treadmills minimum increment capability is 0.1 km·h^−1^. However for logistical and ethical reasons, volunteers subjectively nominated the treadmill running speed they perceived approximated their personal maximal tolerable effort, with the knowledge the third and final effort was the last opportunity to determine a “maximal” effort. The initial speeds were based on the initial speeds they ran at, which were based on the 5 km time trial and final speed of the ${\dot{\text{V}}\text{O}}_{2\max}$ test. Unfortunately due to technical malfunction, blood lactate concentration changes during the incremental test to determine lactate threshold could not be analysed, although we believe the best gauge of maximal constant-rate effort is ultimately determined from actual time trial performance. Hence, 5 km was chosen as the time trial distance as it was estimated to be completed in approximately 20 minutes. The mean time of the 5 km time trial completed was 21 min and 24 seconds.

## 5. Conclusion

The primary aim of this paper is to contribute to the knowledge of the most effective training regimens athletes should embrace to optimise improvements in 5 km run performance. It is acknowledged to address this question further research needs to compare the effects of training strategies over time. Our data indicates that constant-rate running at lactate threshold should be considered worthy of inclusion in investigations as it imposes an identical aerobic metabolic load as interval running over the duration of a time-matched training bout. Another interesting finding is that constant-rate running at lactate threshold allows more distance to be covered and is therefore a more economic training strategy if covering distance is the goal.

### 5.1. Practical Applications

- The similar mean ${\dot{\text{V}}\text{O}}_{2}$ between constant rate at lactate threshold and interval runs indicates that both training strategies may be equally effective in stimulating physiological adaptation and enhancing run performance.
- Constant rate at lactate threshold running will allow athletes to cover 10% further distance in 20 minutes compared to interval running.

## Figures and Tables

**Table 1:** Mean average $\dot{\text{V}}{\text{O}}_{2}$ (mL·min^−1^), $\dot{\text{V}}{\text{O}}_{2}/{\dot{\text{V}}\text{O}}_{2\max}$ (%), and distance covered (metres) for the three treatments with 95% confidence intervals.

	Interval	Submaximal constant rate	Constant rate at lactate threshold
Mean average $\dot{\text{V}}$O_2_	3451 (3269, 3633)^†^	3141 (2969, 3314)^∗*∧*^	3464 (3285, 3643)^†^
$\dot{\text{V}}$O_2_/$\dot{\text{V}}$O_2max_ (%)	83 (79, 88)^†^	76 (72, 80)^∗*∧*^	84 (80, 89)^†^
Distance covered (metres)	4070 (3831, 4309)	4070 (3831, 4309)	4470 (4202, 4737)^∗†^

*P < 0.05 versus respective value in the interval run.

^†^ *P* < 0.05 versus respective value in submaximal constant rate run.

^*∧*^ *P* < 0.05 versus respective value in constant rate at lactate threshold run.
